# Exploring the impact of chronic high‐altitude exposure on visual spatial attention using the ERP approach

**DOI:** 10.1002/brb3.944

**Published:** 2018-03-25

**Authors:** Delong Zhang, Hailin Ma, Jiaqun Huang, Xinjuan Zhang, Huifang Ma, Ming Liu

**Affiliations:** ^1^ Center for the Study of Applied Psychology Key Laboratory of Mental Health and Cognitive Science of Guangdong Province School of Psychology South China Normal University Guangzhou China; ^2^ Plateau Brain Science Research Center South China Normal University/Tibet University Guangzhou China; ^3^ Institute for Brain Research and Rehabilitation South China Normal University Guangzhou China; ^4^ Department of Primary Education Lhasa Normal College Tibet China

**Keywords:** attention, chronic high‐altitude exposure, cognitive resource, event‐related potentials, visual search task

## Abstract

**Introduction:**

Previous studies have reported the slowing of reaction times to attentionally demanding tasks due to a reduction in cognitive resource as a result of chronic high‐altitude exposure. However, it is still largely unknown whether this reaction slowness can be attributed to the attentional allocation change and/or response patterns.

**Methods:**

To clarify this issue, this study investigated attention‐related (N2pc and N2 cc) and response‐related (MP and RAP) event‐related potentials (ERPs) to identify the performance of a visual search task by individuals who had lived in high‐altitude areas for three years compared with those living at sea level.

**Results:**

This study showed that the reaction times in response to a visual search task were significantly longer in the high‐altitude subjects than in the sea level subjects. Corresponding to this behavioral observation, we found a significantly lower N2pc amplitude and a larger N2 cc amplitude in the high‐altitude subjects, suggesting a reduction in spatial attention allocation to the target (N2pc) in these subjects, indicating they need to work harder to preclude cross‐talk between response selection and attention direction (N2 cc). Moreover, we also discovered higher MP amplitudes and longer RAP latencies in the high‐altitude subjects, which further indicated that these subjects were slower and required greater cortical activation while preparing and executing correctly selected responses (MP and RAP).

**Conclusion:**

Nevertheless, this study collectively provided new insights into the attention reaction slowness from high‐altitude exposure.

## 1 INTRODUCTION

Human attentional abilities are sensitive to high altitudes (Lalan Thakur, Anand, & Panjwani, 2011). Many previous studies have indicated that attentional functions are affected by high‐altitude exposure (Virués‐Ortega, Buela‐Casal, Garrido, & Alcázar, 2004; Yan, 2014). High‐altitude exposure was shown to slow the reaction times (RTs) of visual attention tasks (Stivalet et al., 2000; Wang et al., 2014), which may be attributed to a reduction in cognitive resources (Wang et al., 2014). Note that visual attention is a complex mental process that includes the allocation of attention to a target, target selection, and response preparation processes. These processes all contribute to the RT recorded during attentional task execution. Therefore, it is necessary to explore whether the slowing of response efficiency may be attributed to deficiencies in attentional resources to a target, and/or to deficient response performance. The combination of the visual search paradigm and event‐related potential (ERP) method provides an avenue to solve the questions stated above. Visual attention is not only a signal enhancement (i.e., facilitation) process, as proposed by a sensory gain model as a gain control, (Di & Martinez AHillyard, 2003; Hillyard, Vogel, & Luck, 1998; Mangun, 1995; Navalpakkam & Laurent, 2007) but also a suppressive mechanism that may contribute to visual selective attention (Couperus & Mangun, 2010). During visual selective attention, top‐down control may contribute to direct attention to relevant stimuli while simultaneously facilitating suppression of the processing of irrelevant stimuli (Couperus & Mangun, 2010). The visual search task, which requires subjects to find a predefined target stimulus (i.e., presented in arrays randomly including a variable number of bilateral distractor stimuli and the target differs from distractors in orientation change), is usually used to explore visual selective attention, in which the subjects are instructed to press a preassigned button to indicate whether the target is missing in each array of stimuli. To complete the visual search task, several mental processes, such as focusing on a target and the selection and preparation of motor responses to correctly detect the target, should be required. Regarding the attentional processes, previous studies have shown that the ERP component, N2pc (N2‐posterior‐contralateral) (Luck & Hillyard, 1994), is an electrophysiological mark that embodies the visuospatial attention focusing on a target stimulus that supports visual search (Luck & Ford, 1998; Luck & Hillyard, 1994; Woodman & Luck, 1999). The N2pc component was found to be primarily generated in lateral occipital–temporal regions (Hopf, Boelmans, Schoenfeld, Heinze, & Luck, 2002; Lorenzo‐López et al., 2011) with a maximum amplitude over parietal‐occipital electrode sites contralateral to the attended item (Eimer, 1996; Luck & Hillyard, 1994). There are three main theories to interpret the N2pc that is yielded during selective attention processing. The spatial filtering model proposed that target recognition occurs through suppressing distractors around the target (Luck, Girelli, Mcdermott, & Ford, 1997; Luck & Hillyard, 1994). The target enhancement theory posited the opposite of the spatial filtering model, stating that N2pc is more likely reflects the neural process of choice‐processing of task‐related stimuli, which is a process more controlled by a top‐down neural mechanism that is sensitive to task‐related features, rather than the suppression of distractors around the target (Eimer, 1996). In contrast to the two theories above, the parallel mechanism theory proposed that the attentional system involved in visual search is a parallel mechanism in which, on the one hand, the attention system would invoke cognitive resources in line with the current task and process the potential target according to the target's features and on the other hand would restrict the cognitive resources to allocate resources to the distractors during external stimuli processing, by which the attentional system would be more efficient and conducive in processing the current task‐related target to complete the current task (Hickey, Di, & Mcdonald, 2009).

In addition, to make sure that the spatial attention direction does not bias the response selection (Praamstra, 2006; Praamstra & Oostenveld, 2003), the N2 cc component, which exhibits the same polarity and latency as the N2pc component in the visual search task, was observed to be related to the prevention of a manual‐response selection hinging on the target stimulus location in the visual field during visuospatial attention tasks (Oostenveld, Praamstra, Stegeman, & Van, 2001; Praamstra, 2006; Praamstra & Oostenveld, 2003). The N2 cc component was recorded at central electrodes contralateral to the side of the presentation of the target stimuli during visuospatial attention tasks. In visual search tasks, when the target stimulus appears randomly in the right or left visual field, the subjects need to respond with the appropriate hand, whereas other hand is required to respond when the target stimulus is absent. Thus, the subject's responses to the target stimuli are based on the task instructions and are independent of the target's position in the visual field, which can be considered as an executive inhibiting the vulnerability of the response choice of the attentional direction. In this regard, measuring the N2 cc component makes it possible to explore the effects of high altitudes on this executive function during visual search tasks.

As to the movement selection and preparation processes involved in responding to a stimulus, several associated ERP components are generated in the motor cortical areas before a correct response is overtly executed. The readiness potential (RP) is one of the most prominent ERPs and is related to active limb movements (Bötzel, Ecker, & Schulze, 1997; Deecke, Scheid, & Kornhuber, 1969; Shibasaki & Hallett, 2006), in which the motor potential (MP), in the contralateral motor cortex, indicates motor generation of a selected response (Böcker, Brunia, & Cluitmans, 1994); during response execution the reafferent potential (RAP) is associated with sensory‐motor integration processes (Bötzel et al., 1997; Seiss et al., 2002; W Szurhaj & Derambure, 2006). Therefore, measurements of the MP and RAP components will help in examining motor generation and sensory‐motor integration processes related to the corrected response of the visual search task.

By combining the visual search task and ERP components, our previous study reported slowed RTs of an attention task under a high‐load condition in subjects with exposure to high altitudes and that the cognitive resource responding to task demands were reduced (Wang et al., 2014). This study aimed to investigate the basic properties of human visual selective attention as affected by chronic high‐altitude exposure by combining the ERP and visual search paradigms. To this end, healthy and young people who were born and raised in sea level areas but who were then exposed to a high altitude for three years in Tibet were recruited. Focusing on this population, we applied the visual search paradigm to investigate whether the allocation of attention on targets and/or the motor selection/preparation process contribute to RTs recorded during attentional task execution may be affected by chronic high‐altitude exposure.

## 2 METHODS

### 2.1 Subjects

We recruited 20 right‐handed, healthy college students (10 females, 22.65 ± 1.14 years, range 21–25 years) for this study. All these students (altitude of birthplace: 134.45 ± 170 m above sea level) had resided at a high altitude (Tibet, China at 3680 m above sea level) for 3 years prior to being sampled and returned to low altitudes and stayed there <2.5 months each year; they were recruited from Tibet University. In addition, this study also recruited 22 matched control subjects from Ningbo University (Ningbo city, Zhejiang province, China at 4.6 m above sea level) (11 females, 23.09 ± 0.92 years, range: 20–25 years, altitude of birthplace: 97.36 ± 197 m above sea level). The normal control subjects all were born and residing at sea level and had never been to the Qinghai‐Tibet Plateau. Of note, the ERP data of the high‐altitude residents were collected at Tibet University, and those of the sea level altitude residents were acquired at Ningbo University; the instrument model and the laboratory environment were the same in the two laboratories in each of the two cities (Lhasa and Guangzhou), and the experiment was performed by the same experimenters. The national examinations for college entrance scores of the subjects were matched between the two groups. All subjects’ vision was normal or corrected‐to‐normal. None of the subjects had a history of neurological/psychiatric illnesses, major medical illness, or use of medications that influenced cognition. Three subjects from the high‐altitude group were excluded, and one participant was excluded from the other group due to frequent eye movements or excessive artifacts. All subjects signed informed consent forms and were paid (¥40/hour). The study adhered to the guidelines of the Declaration of Helsinki and was approved by the Government of Tibet Autonomous Region in this study.

### 2.2 Materials and Stimuli

**Figure 1:**
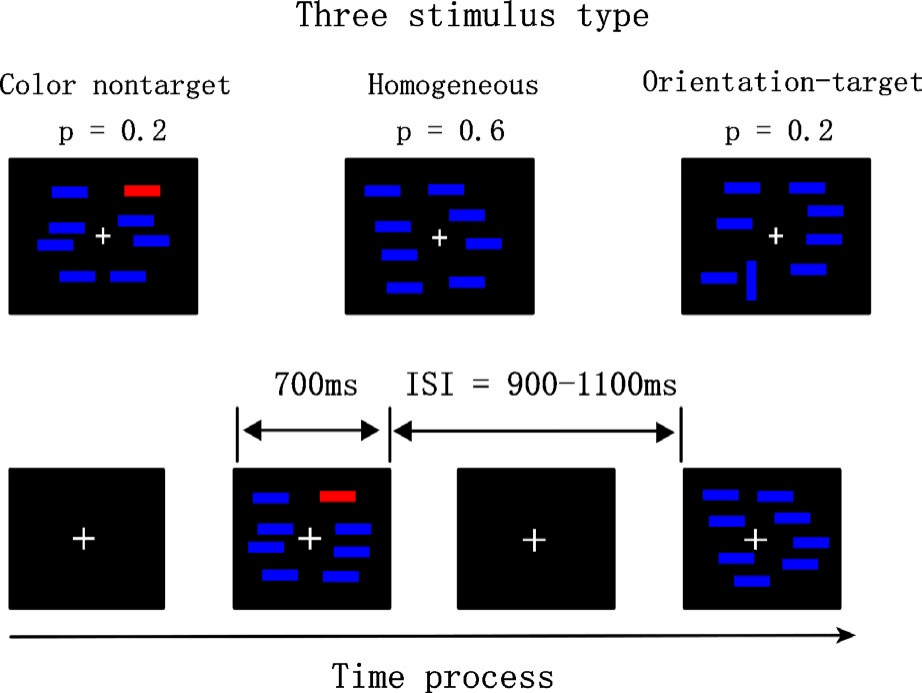
Graphic description of task properties

The laboratory was electrically shielded, quiet, and dim. The distance between each subject and the computer screen was approximately 100 cm. There were three types of search arrays (i.e., the homogeneous arrays, orientation‐target arrays, and color nontarget arrays), which were displayed on the screen against a black background, and a white cross served as constant, visible fixation. Of note, the homogeneous arrays (*p *=* *.6) were composed entirely of 8 small, blue (RGB 0, 0, 255), horizontal, and identical bars (four on each side of the fixation). The orientation of the target arrays (*p *=* *.6) contained seven blue‐horizontal identical bars with one blue‐vertical bar. The orientation‐target was a target (the vertical bar) presented among distractors, specifically a vertical bar among horizontal distractors (four on each side of fixation). Colored, nontarget arrays (*p *=* *.2) contained seven blue‐horizontal bars and one red‐horizontal (RGB255, 0, 0) bar. The orientation of the target and the colored nontargets were equally likely to be randomly appear on the left or right visual hemifield of the fixation point. The three array types were randomly presented, and within each array, the positions of the bars also randomly varied (Figure 1). The stimuli were placed randomly within an imaginary rectangle, which subtended the visual angle of 9.2 × 6.9 degrees and was centered at a constantly visible fixation point. Each singular bar subtended 0.3 × 0.9 degrees.

### 2.3 Procedure

All the stimuli were presented and controlled using E‐prime 2.0. Each trial started with a fixation cross at the center of the screen that stayed visible for a randomly varying duration (900–1100 ms), which was visible throughout the remainder of the trials before the search pattern appeared. The fixation cross was then replaced by a search array for a fixed duration of 750 ms. The 750‐ms‐search array was also presented at the center of computer screen. The trial sequences were presented in a random order. The same orientation was defined for the targets through all trials, and the subjects were not informed of the color nontargets beforehand. Each subject performed one practice block of 64 trials (including three types of search arrays), followed by six experimental blocks of 250 trials. Each block contained at least 10 arrays consisting of one orientation target and 10 arrays including a color nontarget presented to every hemifield and no <80 homogeneous arrays. Each trial was started by pressing the “F” and “J” keys simultaneously on the keyboard with the right and left index fingers, respectively. Subjects were instructed to indicate if the target was displayed in each search array by pressing one of two buttons as rapidly and accurately as possible. Subjects responded with one hand for the presence of the orientation‐target arrays (target‐present) and with the other hand for the presence of the homogeneous arrays and color nontarget arrays (target‐absent); the hand assignment was offset across subjects.

### 2.4 ERP recording

The electroencephalography (EEG) data were recorded by a NeuroScan Synamps‐2 that employed Ag/AgCl electrodes placed at 64 standard locations in accordance with the extended international 10–20 system and a precabled electrode cap. The online reference electrode was placed on the left mastoid during recordings and the EEG was rereferenced offline to the right mastoid for data analysis. All the active electrodes were grounded with GND. The horizontal electrooculogram (HEOG) was employed to measure horizontal eye movements, which were recorded using the voltage difference between the electrodes placed lateral to the external canthi. The vertical electrooculogram (VEOG) was used to detect eye blinks, which were recorded using the voltage difference between two electrodes placed above and below the left eye. The EEG signal was filtered online with a bandpass of 0.05–200 Hz and was sampled at 500 Hz. Of note, the impedances were maintained below 20 kΩ.

### 2.5 Data analysis

#### 2.5.1 Behavioral data

For every subject, the RTs were recorded online to the three types of search arrays in each block (as for the orientation target and the color non‐target arrays, the RTs to stimuli that appeared in the right/left hemifield were recorded, respectively). Trials with missing or incorrect responses were precluded from the behavioral analysis. The percentage of correct trials was computed for all conditions. To better meet the assumptions of normality and homogeneity of variance, the hit rates were arcsine‐square root transformed before the analyses. Repeated‐measures ANOVAs were employed to test the differences in the mean correct RTs related to the experimental conditions across the groups. There were two between‐subjects factors, namely altitude (i.e., sea level, high altitude) and response hand (i.e., right, left), and there was one within‐subjects factor (search array: homogeneous, orientation right‐left target, color right‐left nontarget). The hit rates, both original and transformed, were compared across the groups using a mixed design ANOVA, with altitude and response hand as the between‐subjects factors and the search array as the within‐subjects factor.

#### 2.5.2 EEG data analysis

NeuroScan software (Curry 7.0) was used to analyze all EEG data. The EEG data were filtered digitally offline with a 0.1–30 Hz band‐pass filter. Epochs were set between −100 and 900 ms related to the stimulus presentation to acquire attention‐related ERPs (N2pc and N2 cc, see below). The EEG data were segmented into epochs of 1000 ms postresponse/preresponse for the motor‐related RP components (MP and RAP, see below). Epochs exceeding ± 100 μV, such as for blinks, and horizontal or vertical eye movements were excluded from averaging, as well as the epochs related to incorrect or nonresponses. Considering that the ERP effects on the attentional focusing related to color nontarget stimuli in previous studies were not different, the EEG data were averaged separately for the orientation targets occurring in the right/left visual field (RVF/LVF) to acquire the attention‐related ERPs (N2pc and N2 cc) and response‐related RP components (MP and RAP), resulting in four attention‐related and two response‐related waveforms for each subject (see below for details).

##### N2pc and N2 cc

**Figure 2:**
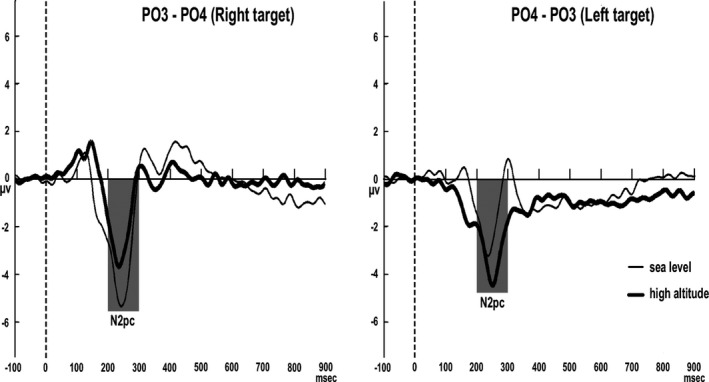
N2pc component. Subtraction waveforms superimposed for sea level and high‐altitude subjects

**Figure 3:**
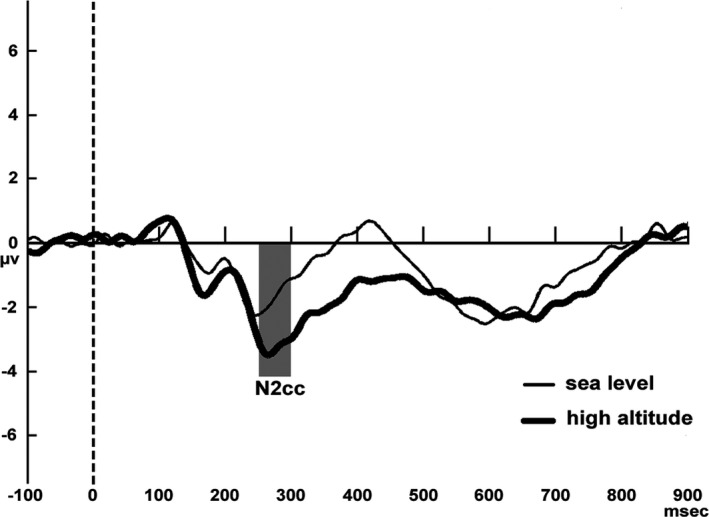
N2 cc component acquired by the formula “N2 cc = [(C3‐C4)RVF attention +  (C4‐C3)LVF attention]/2”.Subtraction waveforms superimposed for sea level and high ‐altitude subjects

The N2pc component was acquired from the contralateral (average of left‐sided electrode with RVF target and right‐sided electrode with LFV target) minus the ipsilateral (average of left‐sided electrode with LVF target and right‐sided electrode with RVF target) difference waves. The procedure for acquiring the N2pc was applied at the posterior electrodes PO3 and PO4. The operation for calculating the N2pc component can be summed up by the formula PO3‐PO4 for the right‐hemifield target stimuli and PO4‐PO3 for the left‐hemifield target stimuli. For the derivation of the N2 cc component, different waveforms for each subject were acquired by subtracting the ipsilateral from the contralateral ERPs associated with the visual field of attention. The operation for acquiring the N2 cc can be summed up by the following formula: [(C3 − C4) _RVF attention_ + (C4 − C3) _LVF attention_]/2. This procedure extracts lateralized potentials, collapsing the activity of both hemispheres. In the sea level and high‐altitude groups, the N2pc and N2 cc components were measured as the mean amplitude from 145 to 300 ms and 266 to 300 ms, respectively, based on inspection of the grand averages (see Figures 2, 3) and resembled the temporal window in previous studies (Amenedo, Lorenzo‐López, & Pazo‐Álvarez, 2012). To test the altitude effects on the N2pc and N2 cc parameters, an independent‐samples *t* test (*p *<* *.05) was carried out on the peak amplitude, mean amplitude, and latency values. The peak amplitude, mean amplitude, and latency values of the N2pc and N2 cc components were entered into separate ANOVAs to test the altitude effects on these components. Finally, mixed ANOVAs were implemented containing one within‐subjects factor, namely electrode (two levels: left, right) and one between‐subjects factor, namely altitude (sea level, high‐altitude).

##### MP and RAP

At relevant electrodes, the RAP averages to the orientation targets were analyzed from the contralateral motor areas (C3 for right‐hand responders, C4 for left‐hand responders). Baseline was defined as a 200‐ms interval between 800 and 1000 ms before the motor response (button press). The latency and amplitude of the RAP and the MP, as the most negative peak in the average ERP, were measured at central electrodes and within the interval between the stimulus response execution for each subject. In addition, after the MP, for the most positive displacement of RP and RAP, the peak latency and amplitude values were detected for the most positive displacement of RP and RAP, after the MP.

Repeated‐measures ANOVAs were employed to test if any differences existed in the peak latency and amplitude values of MP, RAP, and MP onset and rise time values, with altitude (sea level, high‐altitude) and response hand (right, left) as the between‐subjects factors, and the target‐response compatibility (compatible target‐response side, incompatible target‐response side) as the within‐subjects factor. When it was appropriate, the degrees of freedom were corrected by the conservative Greenhouse‐Geisser estimate. Post hoc paired multiple comparisons of the mean values were carried out (with the Bonferroni correction) in situations where the significant effects were revealed by the ANOVAs due to the factors and their interactions. All statistical tests were set at an alpha level of 0.05.

## 3 RESULTS

### 3.1 Behavioral results

As shown in Table 1, RTs were substantially slower in the high‐altitude group for each array type (F(1, 38) = 5.606, *p *<* *.05, $\eta_{p}^{2}$ = .129), regardless of the which hand was assigned to indicate the targets (F(1, 38)  = 0.001, *p* = .971). Additionally, the main effect of array type on the mean RTs was significant (F(4, 152)  = 75.482, *p *<* *.001, $\eta_{p}^{2}$ = .665), showing that the longest RTs were related to the orientation targets, the intermediate RTs to the color nontargets, and the shortest RTs to the homogeneous arrays (see Table 2). Interactions were nonsignificant between altitude and response hand (F(1, 38)  = 0.328, *p* = .570), between altitude and array type (F(4, 152)  = 0.117, *p* = .976) and between response hand and array type (F(4, 152)  = 0.986, *p* = .417). Moreover, the target‐response compatibility effects on the RT values were nonsignificant (F(1, 82)  = 0.017, *p* = .897) in both the sea level (target‐response compatible: 501.020 ± 50.507 ms, incompatible: 493.83 ± 47.16 ms) and high‐altitude groups (target‐response compatible: 515.40 ± 52.20 ms, incompatible: 525.42 ± 52.40 ms). The effects of altitude (F(1, 211)  = 2.04, *p* = .155) and response hand (F(1, 211)  = 2.274, *p* = .133) on hit rates (sea level right hand: 94.91 ± 11.19%, left hand: 96.498 ± 5.693%; high‐altitude right hand: 96.42 ± 6.86%, left hand: 97.86 ± 2.91%) were not significant. The interactions were nonsignificant between altitude and response hand (F(1, 211)  = 0.005, *p* = .942). The results of an ANOVA executed on the arcsine root transformed data indicated no significant effects of altitude (F(1, 211)  = 1.795, *p *<* *.182). Neither the main effect of response hand (F(1, 211)  = 0.173, *p* = .678) nor the interactive effect between altitude and response hand (F(1, 211)  = 0.011, *p* = .918) were observed to be significant for these transformed data.

**Table 1:** Age, gender, education, altitude of birth place of each group

	Age (yrs)^a^	Gender (M/F)^b^	Education (yrs)^a^	Altitude (m)^a^
Sea level	22.65 ± 1.14	9/11	15.05 ± 0.51	97.36 ± 197
High altitude	23.09 ± 0.92	12/10	15.14 ± 0.56	134.45 ± 170
*t /* χ^*2*^	−1.387	0.382	−0.520	−0.600
*p*	0.173	0.758	0.606	0.552

M: male, F: female; Altitude: altitude of birth place.

a Independent sample *t* test.

b chi‐square test.

**Table 2:** Mean values (standard deviation) of RTs (ms) across array types and assigned response hand in the two groups

	Homogeneous	Color Right	Color Left	Target Right	Target Left
Right hand					
Sea level	413.40 (36.180)	426.27 (43.342)	422.66 (39.196)	493.25 (45.598)	479.53 (43.554)
High altitude	420.40 (27.968)	429.35 (31.782)	426.75 (27.222)	513.75 (63.247)	525.46 (64.476)
Left hand					
Sea level	391.92 (26.531)	399.67 (31.382)	399.24 (30.800)	508.13 (48.213)	508.80 (56.079)
High altitude	428.30 (51.594)	434.03 (49.553)	434.69 (48.263)	524.25 (43.930)	518.50 (44.912)

### 3.2 Electrophysiological results

#### 3.2.1 N2pc and N2 cc components

For the N2pc peak amplitude, the main effects of altitude (F(1, 40)  = 1.930, *p* = .172) and sites (F(1, 40)  = 0.596, *p* = .445) were not significant. The interactive effect between altitude and sites was marginal significant (F(1, 40)  = 2.863, *p* = .098, $\eta_{p}^{2}$ = .067), with the peak amplitude for the sea level group being more negative than that for the high‐altitude group with the right‐hemifield target stimulus (F(1, 40)  = 5.148, *p* = .029, $\eta_{p}^{2}$ = .114). For the N2pc peak latency, no significant main or interactive effect was found (*ps*. > 0.05) (see Table 3 and Figure 2). For the N2 cc mean amplitude, the main effect of altitude (*t* = 2.074, *p* = .045) was significant, with the mean amplitude of the N2 cc component for the high‐altitude group being more negative than that for the sea level group (see Table 3 and Figure 3).

**Table 3:** Mean values (standard deviation) of N2pc and N2 cc components in the two groups

	N2pc (right target)		N2pc (left target)		N2 cc	
	Sea level	High altitude	Sea level	High altitude	Sea level	High altitude
Peak latency (ms)	247.18 (36.54)	228.29 (33.88)	231.27 (37.33)	235.62 (35.46)	284.64 (55.04)	282.38 (51.65)
Peak amplitude (μV)	−6.68 (3.41)	−5.11 (2.60)	−5.07 (3.50)	−5.27 (2.32)	−4.16 (2.26)	−4.33 (2.24)
Mean amplitude (μV)	−2.46 (2.33)	−1.50 (1.49)	−1.54 (2.80)	−2.40 (2.00)	−0.97 (2.04)	−2.17 (2.13)

The mean amplitude was measured by calculating mean amplitude value of choiced temporal windows (N2pc: 145 ms–300 ms; N2 cc: 266 ms–300 ms),and peak amplitude was measured by calculating the bigger amplitude value of choiced temporal windows (N2pc: 145 ms–300 ms; N2 cc: 266 ms–300 ms).

#### 3.2.2 MP and RAP components

For the MP amplitude, the main effect of altitude was significant (F(1, 38)  = 5.041, *p* = .031, $\eta_{p}^{2}$ = .117), with a larger MP amplitude in the high‐altitude group than in the sea level group. The main effect of target‐response compatibility was significant (F(1, 38)  = 24.622, *p* = .000, $\eta_{p}^{2}$ = .393), with a larger amplitude in the target‐response incompatibility than in the target‐response compatibility. The main effect of response hand was not significant (F (1, 38)  = 0.110, *p* = .742). The interactive effect between altitude and response hand was not significant (F(1, 38)  = 0.981, *p* = .328), but there was a larger amplitude for the high‐altitude group than that for the sea level group when responding to the target with their right hand (F(1, 38)  = 5.525, *p* = .024, $\eta_{p}^{2}$ = .127), although no significant difference was found between the two groups when responding to the target with their left hand (F(1, 38)  = 0.748, *p* = .393). The interactive effect between altitude and target‐response compatibility was not significant (F(1, 38)  = 1.225, *p* = .275). The interactive effect between target‐response compatibility and response hand was significant (F(1, 38)  = 16.833, *p* = .000, $\eta_{p}^{2}$ = .393), with a larger amplitude for target‐response incompatibility than that for target‐response compatibility with the left hand (F(1, 38)  = 39.032, *p* = .000, $\eta_{p}^{2}$ = .507), but no significant difference was found between the two target‐response compatibilities when responding to the target with the right hand (F(1, 38)  = 0.390, *p* = .536). For the MP latency, the main effect of target‐response compatibility was significant (F(1, 38)  = 6.456, *p* = .015, $\eta_{p}^{2}$ = .145), with a longer latency in the target‐response incompatibility than in the target‐response compatibility. No other main effects or interactive effects were found (*ps*. > 0.05).

**Figure 4:**
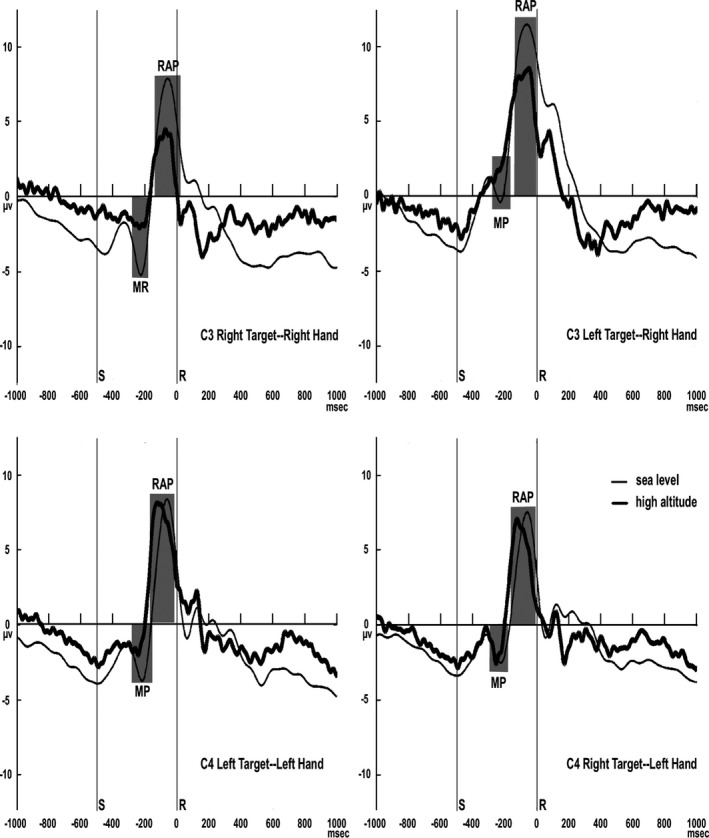
Response‐related ERPs (MP and RAP) in the two groups. Top and middle panels: target‐response compatibility effects for right and left‐hand responders. Lower panel: assigned hand effects shown for compatible trials. S: target stimulus onset. R: response execution (button pressure). The waveforms were low‐pass filtered at 10 Hz for visualization purposes only

The RAP peak amplitude (F(1, 38)  = 0.053, *p* = .820) was not affected by altitude (Table 4 and Figure 4). The RAP peak amplitude (F(1, 38)  = 0.201, *p* = .656) was also not affected by the response hand (Table 3). For the amplitude of RAP, no significance was observed in the target‐response compatibility effects (F(1, 38)  = 10.531, *p* = .002, $\eta_{p}^{2}$ = .217). Finally, the significant effects of altitude (F(1, 38) = 5.177, *p *=* *.029, $\eta_{p}^{2}$ = .120) were observed on the latency of the RAP component, with a longer latency for the high‐altitude group than for the sea level group. Regarding the latency of RAP, both the effects of the response hand (F(1, 39) = 0.861, *p *=* *.359) and the target‐response compatibility (F(1, 39) = 0.879, *p *= .354) were nonsignificant and no interactions were found among any of the factors (*ps. *> 0.05).

**Table 4:** Mean amplitude and latency values (standard deviation) of RP components at the contralateral electrode (C3 for right hand; C4 for left hand) across assigned response hand in each group

Hand	Altitude group	RP component		Compatible target	Incompatible target
Right	Sea level	MP	Peak amplitude (μV)	−0.34 (3.30)	3.62 (3.66)
			Peak latency (ms)	−274.91 (79.78)	−240.36 (73.75)
		RAP	Peak amplitude (μV)	7.45 (5.90)	11.26 (6.27)
			Peak latency (ms)	−63.09 (25.73)	−65.27 (28.74)
	High altitude	MP	Peak amplitude (μV)	1.76 (2.89)	4.71 (3.55)
			Peak latency (ms)	−276.60 (72.94)	−201.40 (37.88)
		RAP	Peak amplitude (μV)	8.11 (5.46)	11.57 (4.29)
			Peak latency (ms)	−78.80 (57.80)	−82.00 (52.07)
Left	Sea level	MP	Peak amplitude (μV)	−0.12 (3.30)	2.04 (3.70)
			Peak latency (ms)	−239.45 (62.67)	−252.36 (64.45)
		RAP	Peak amplitude (μV)	9.90 (2.99)	8.97 (3.95)
			Peak latency (ms)	−66.55 (28.48)	−67.64 (31.29)
	High altitude	MP	Peak amplitude (μV)	3.74 (4.67)	3.80 (4.91)
			Peak latency (ms)	−214.18 (62.88)	−218.73 (65.50)
		RAP	Peak amplitude (μV)	10.50 (5.53)	8.85 (6.16)
			Peak latency (ms)	−95.45 (38.02)	−101.64 (39.40)

## 4 DISCUSSION

When several objects strive for attention in visual environments, finding relevant information and ignoring unrelated objects and events becomes challenging. To accomplish the above mentioned visual processes, attention allocation to a target, motor selection and preparation processes need to occur during attention. An interesting issue is how the attentional process changes in response to a reduction in attentional resources as a result of high‐altitude exposure. In the present study, the stimulus‐ and response‐related ERPs were recorded in response to a visual search task for a single feature target clarified by an orientation change associated with bilateral surrounding distractor stimuli in subjects who immigrated to a high‐altitude area (3680 m) and those who have lived in a sea level area. Our previous study results indicated a slowing of RTs to an attentional task in the immigrants to the high‐altitude area (Wang et al., 2014). The present study aimed to further identify whether the attentional reaction slowness due to chronic high‐altitude exposure was associated with deficits in attentional selection and/or motor selection/preparation, as indexed by the N2pc, N2 cc, and RP components, respectively. The behavioral performance analyses and simultaneous EEG recordings revealed remarkable differences between the sea level and high‐altitude groups. The results demonstrated the existence of RT difference between the two groups, with the high‐altitude group showing abnormally longer RTs, an effect that was not observed on hit rates. Regarding the N2pc peak amplitude, the high‐altitude group showed a smaller amplitude in response to the right‐hemifield target stimulus than that of the sea level group. Instead, regarding the N2 cc component mean amplitude, the high‐altitude group exhibited a larger amplitude than that of the sea level group. Additionally, we found that the MP amplitude in the high‐altitude group was larger than that in the sea level group and the latency of the RAP component for the high‐altitude group was longer than that for the sea level group.

The behavioral results suggested an altitude‐related delay in RTs, which was consistent with our previous findings (Wang et al., 2014), and some behavioral studies proposed that the responses of subjects who have had chronic high‐altitude exposure are slower in visual search tasks than those of subjects who were living at sea level (Stivalet et al., 2000). It should be noted that, regarding hit rates, there was no main effect of altitude once the original data were arcsine root transformed, which was inconsistent with the findings of a previous study (Wang et al., 2014). One of the most possible interpretations of this finding may be that there exists a ceiling effect in the hit rates in which some high‐altitude subjects might have a trade‐off between the accuracy and reaction speed, while other subjects perform equally well with the sea level subjects.

As mentioned above, the N2pc component is a well‐established electrophysiological index that reflects visuospatial attentional shifts to target stimuli (Hopf, Boelmans, Schoenfeld, Luck, & Heinze, 2004; Lorenzo‐López et al., 2011). The present results found that the N2pc peak amplitude for the sea level subjects was more negative than that for the high‐altitude subjects with a right‐hemifield target stimulus, which provided evidence that the high‐altitude subjects distribute less attentional resources to the target stimulus’ location during visual search. It should be noted that this result was in line with the findings of a previous study that focused on aging (Amenedo et al., 2012).

The N2 cc is an attention‐related motor component that reflects the inhibition or precluding of incorrect response emission promoted by targets (Praamstra, 2006; Praamstra & Oostenveld, 2003), which is considered to play an important role in the executive control system in ensuring correct and flexible responding (Miller & Cohen, 2001; Ridderinkhof, Wp, Segalowitz, & Carter, 2004). In this study, a larger N2 cc peak amplitude was observed in the high‐altitude subjects rather than the sea level individuals, indicating a trend of higher premotor activation to prevent incorrect responses. Previous studies found a trend of lower N2pc‐like and higher N2 cc‐like amplitudes in an older group than in young adults (Amenedo et al., 2012; Lubbe & Rolf, 2002), and the same results were also observed in Parkinson`s Disease (Praamstra & Plat, 2001). In the present study, a lower amplitude of the N2pc component was observed in the high‐altitude group, along with a trend of higher amplitudes in the N2 cc component. Based on the observations above, we speculated that during the visual search task, high‐altitude exposure may induce poor attentional sensory‐motor mapping, which may promote the selection of responses ipsilateral to the targets’ location individually of being the stimulus‐related to the correct response (Lubbe, 2002).

The MP associated with the preparation and initiation of motor command leading to an overt response is generated in the contralateral primary motor cortex (Böcker et al., 1994; Shibasaki & Hallett, 2006). This study found that the motor MP component showed higher peak amplitudes in the high‐altitude subjects than that in the sea level group, which was independent of the hand assigned to answer the target and the target‐response compatibility effects. Similar findings were also found in previous studies of the aging population (Amenedo et al., 2012; Falkenstein, Yordanova, & Kolev, 2006). The altitude‐related change in the MP component indicated that high‐altitude individuals may need higher activation of the contralateral motor cortex to correctly prepare and implement selected responses to the targets to meet the demands of the visual search task.

The result regarding the motor RAP component showed a longer latency in the high‐altitude subjects, which was independent of the hand assigned to answer the target and the target‐response compatibility effects. The RAP component is associated with different functional levels of facilitation of muscles and joints and is a component that is responsible for the movement to motor cortico‐spinal cells and for controlling ongoing movement that is needed (William Szurhaj et al., 2005; W Szurhaj et al., 2006). Some researchers have noted that longer RAP latencies suggest that the sensory‐motor integration is flawed; thus, during the execution of correct responses in a visual search task, a poorer control of movement occurs (Amenedo et al., 2012; Bötzel et al., 1997).

In the present study, the main effect of the hand chosen to respond to targets was not significant on the peak latency or the amplitude of the MP or RAP. However, there a larger MP amplitude was found for the high‐altitude group than for the sea level group when the subjects responded to the target with their right hand. However, no significant difference was found with their left hand. In addition, the interactive effect between the target‐response compatibility and the response hand was significant, with a larger MP amplitude in the target‐response incompatibility than in the target‐response compatibility with the left hand, but no significant difference was observed for the right hand. These results indicated that, during response execution, the sensory‐motor integration and motor preparation were affected by high‐altitude exposure when the movements were performed with the dominant hand.

The biggest contribution of the present study may lie in the fact that we refined the neurophysiological basis underlying the attentional reaction slowness as a result of high‐altitude exposure. In addition, the main findings consistently showed that the high‐altitude‐related behavioral slowing observed in visual search tasks were caused not only by the slower correct responses to detected targets but also on account of the following: (a) slower activation in the dorsolateral premotor cortex to suppress incorrect responses promoted by target location while selecting the correct, (b) higher activation patterns in motor areas while correctly preparing for selected responses, and (c) sensory‐motor mapping was poorer during the correct responses execution. More importantly, all the findings related to the neurophysiological basis underlying the attentional reaction slowness were similar to those of aging, which may provide new aspects to understanding the mechanism of cognition changes due to high‐altitude exposure. In addition, the present findings also gave a possible interpretation of the cerebral effects under high‐altitude exposure. It should be noted that the present findings provided new experimental evidence supporting the reasonability of the attentional parallel mechanism opinion stating that appropriate cognitive resource allocation for the current task‐related target to efficiently and conductively complete the current task is very important for the visual attention selection process. In addition, the limitation of cognitive resources resulting from high‐altitude exposure will affect the allocation of cognitive resources during the visual attention selection process, which could be reflected in aspects of the ERP components (e.g., N2pc and N2 cc) and will finally embody attention behavioral performance. Regarding this aspect, an important avenue for avoiding the reduction in attention performance is to maintain the available cognitive resources for the high‐altitude participants. The present finding was derived from the young participants; thus, the main results should be validated in elders in the future works. Of course, although the present study provided experimental evidence for the neurophysiological change related to the attention reaction slowness due to high‐altitude exposure, it is still necessary to combine multimodal neuroimaging datasets (e.g., task fMRI, positron emission tomography, arterial spin labeling, and diffusion tensor imaging) to provide more insights into the neural basis underlying attention reaction slowness due to high‐altitude exposure, and even the neurotransmitter disruption, physiological basis, structural basis, and the effects of high‐altitude exposure on the collective behavior of the brain.

## 5 CONCLUSIONS

The present study explored the attention‐ and response‐related ERP components underlying the reaction slowness in visual search tasks performed by high‐altitude subjects. The present data showed that not only the attention resource allocation to the targets but also the target selection and response preparation processes exhibit deficiencies in responding due to high‐altitude exposure. The present findings provided experimental evidence for the neurophysiological basis underlying the attention reaction slowness in high‐altitude areas.

## COMPETING INTEREST

The authors declare no competing financial interests.

## References

[brb3944-bib-0001] Amenedo, E. , Lorenzo‐López, L. , & Pazo‐Álvarez, P. (2012). Response processing during visual search in normal aging: The need for more time to prevent cross talk between spatial attention and manual response selection. Biological Psychology, 91(2), 201–211. 22743592 10.1016/j.biopsycho.2012.06.004

[brb3944-bib-0002] Böcker, K. B. E. , Brunia, C. H. M. , & Cluitmans, P. J. M. (1994). A spatio‐temporal dipole model of the readiness potential in humans: i. Finger movement. Electroencephalography & Clinical Neurophysiology, 91(4), 286–294. 7523078 10.1016/0013-4694(94)90192-9

[brb3944-bib-0003] Bötzel, K. , Ecker, C. , & Schulze, S. (1997). Topography and dipole analysis of reafferent electrical brain activity following the Bereitschaftspotential. Experimental Brain Research, 114(2), 352–361. 9166924 10.1007/pl00005643

[brb3944-bib-0004] Couperus, J. W. , & Mangun, G. R. (2010). Signal enhancement and suppression during visual‐spatial selective attention. Brain Research, 1359(1359), 155. 20807513 10.1016/j.brainres.2010.08.076 PMC2955768

[brb3944-bib-0005] Deecke, L. , Scheid, P. , & Kornhuber, H. H. (1969). Distribution of readiness potential, pre‐motion positivity, and motor potential of the human cerebral cortex preceding voluntary finger movements. Experimental Brain Research, 7(2), 158–168. 5799432 10.1007/BF00235441

[brb3944-bib-0006] Di, R. F. , & Martinez AHillyard, S. A. (2003). Source analysis of event‐related cortical activity during visuo‐spatial attention. Cerebral Cortex, 13(5), 486. 12679295 10.1093/cercor/13.5.486

[brb3944-bib-0007] Eimer, M. (1996). The N2pc component as an indicator of attentional selectivity. Electroencephalography & Clinical Neurophysiology, 99(3), 225–234. 8862112 10.1016/0013-4694(96)95711-9

[brb3944-bib-0008] Falkenstein, M. , Yordanova, J. , & Kolev, V. (2006). Effects of aging on slowing of motor‐response generation. International Journal of Psychophysiology Official Journal of the International Organization of Psychophysiology, 59(1), 22–29. 16257076 10.1016/j.ijpsycho.2005.08.004

[brb3944-bib-0009] Hickey, C. , Di, L. V. , & Mcdonald, J. J. (2009). Electrophysiological indices of target and distractor processing in visual search. Journal of Cognitive Neuroscience, 21(4), 760–775. 18564048 10.1162/jocn.2009.21039

[brb3944-bib-0010] Hillyard, S. A. , Vogel, E. K. , & Luck, S. J. (1998). Sensory gain control (amplification) as a mechanism of selective attention: Electrophysiological and neuroimaging evidence. Philosophical Transactions of the Royal Society of London, 353(1373), 1257. 9770220 10.1098/rstb.1998.0281 PMC1692341

[brb3944-bib-0011] Hopf, J. M. , Boelmans, K. , Schoenfeld, A. M. , Heinze, H. J. , & Luck, S. J. (2002). How does attention attenuate target‐distractor interference in vision?. Evidence from magnetoencephalographic recordings. Cognitive Brain Research, 15(1), 17–29. 12433380 10.1016/s0926-6410(02)00213-6

[brb3944-bib-0012] Hopf, J. M. , Boelmans, K. , Schoenfeld, M. A. , Luck, S. J. , & Heinze, H. J. (2004). Attention to features precedes attention to locations in visual search: Evidence from electromagnetic brain responses in humans. Journal of Neuroscience the Official Journal of the Society for Neuroscience, 24(8), 1822–1832. 10.1523/JNEUROSCI.3564-03.2004 PMC6730400 14985422

[brb3944-bib-0013] Lalan Thakur, K. R. , Anand, J. P. , & Panjwani, U. (2011). Event related potential (ERP) P300 after 6 months residence at 4115 meter. Indian Journal of Medical Research, 134(1), 113. 21808143 PMC3171904

[brb3944-bib-0014] Lorenzo‐López, L. , Gutiérrez, R. , Moratti, S. , Maestú, F. , Cadaveira, F. , & Amenedo, E. (2011). Age‐related occipito‐temporal hypoactivation during visual search: relationships between mN2pc sources and performance. Neuropsychologia, 49(5), 858–865. 21237184 10.1016/j.neuropsychologia.2011.01.015

[brb3944-bib-0015] Lubbe, R. H. J. , & Rolf, V. (2002). Aging and the Simon task. Psychophysiology, 39(1), 100–110. 12206290 10.1017/S0048577202001221

[brb3944-bib-0016] Luck, S. J. , & Ford, M. A. (1998). On the role of selective attention in visual perception. Proceedings of the National Academy of Sciences of the United States of America, 95(3), 825–830. 9448247 10.1073/pnas.95.3.825 PMC33804

[brb3944-bib-0017] Luck, S. J. , Girelli, M. , Mcdermott, M. T. , & Ford, M. A. (1997). Bridging the gap between monkey neurophysiology and human perception: An ambiguity resolution theory of visual selective attention. Cognitive Psychology, 33(1), 64–87. 9212722 10.1006/cogp.1997.0660

[brb3944-bib-0018] Luck, S. J. , & Hillyard, S. A. (1994). Spatial filtering during visual search: Evidence from human electrophysiology. Journal of Experimental Psychology Human Perception & Performance, 20(5), 1000–1014. 7964526 10.1037//0096-1523.20.5.1000

[brb3944-bib-0019] Mangun, G. R. (1995). Neural mechanisms of visual selective attention. Psychophysiology, 32(1), 4. 7878167 10.1111/j.1469-8986.1995.tb03400.x

[brb3944-bib-0020] Miller, E. K. , & Cohen, J. D. (2001). An integrative theory of prefrontal cortex function. Annual Review of Neuroscience, 24(1), 167. 10.1146/annurev.neuro.24.1.167 11283309

[brb3944-bib-0021] Oostenveld, R. , Praamstra, P. , Stegeman, D. F. , & Van, O. A. (2001). Overlap of attention and movement‐related activity in lateralized event‐related brain potentials. Clinical Neurophysiology Official Journal of the International Federation of Clinical Neurophysiology, 112(3), 477–484. 11222970 10.1016/s1388-2457(01)00460-6

[brb3944-bib-0022] Praamstra, P. (2006). Prior information of stimulus location: Effects on ERP measures of visual selection and response selection. Brain Research, 1072(1), 153–160. 16406014 10.1016/j.brainres.2005.11.098

[brb3944-bib-0023] Praamstra, P. , & Oostenveld, R. (2003). Attention and movement‐related motor cortex activation: A high‐density EEG study of spatial stimulus–response compatibility. Brain Research. Cognitive Brain Research, 16(3), 309–322. 12706212 10.1016/s0926-6410(02)00286-0

[brb3944-bib-0024] Praamstra, P. , & Plat, F. M. (2001). Failed suppression of direct visuomotor activation in Parkinson's disease. Journal of Cognitive Neuroscience, 13(1), 31–43. 11224907 10.1162/089892901564153

[brb3944-bib-0025] Ridderinkhof, K. R. , Wp, V. D. W. , Segalowitz, S. J. , & Carter, C. S. (2004). Neurocognitive mechanisms of cognitive control: The role of prefrontal cortex in action selection, response inhibition, performance monitoring, and reward‐based learning. Brain & Cognition, 56(2), 129–140. 15518930 10.1016/j.bandc.2004.09.016

[brb3944-bib-0026] Seiss, E. , Hesse, C. W. , Drane, S. , Oostenveld, R. , Wing, A. M. , & Praamstra, P. (2002). Proprioception‐related evoked potentials: Origin and sensitivity to movement parameters. NeuroImage, 17(1), 461–468. 12482098 10.1006/nimg.2002.1211

[brb3944-bib-0027] Shibasaki, H. , & Hallett, M. (2006). What is Bereitschaftspotential?. Clinical Neurophysiology, 117(11), 2341–2356. 16876476 10.1016/j.clinph.2006.04.025

[brb3944-bib-0028] Stivalet, P. , Leifflen, D. , Poquin, D. , Savourey, G. , Launay, J. C. , Barraud, P. A. , & Bittel, J. (2000). Positive expiratory pressure as a method for preventing the impairment of attentional processes by hypoxia. Ergonomics, 43(4), 474–485. 10801081 10.1080/001401300184350

[brb3944-bib-0029] Szurhaj, W. , Bourriez, J. L. , Kahane, P. , Chauvel, P. , Mauguière, F. , & Derambure, P. (2005). Intracerebral study of gamma rhythm reactivity in the sensorimotor cortex. European Journal of Neuroscience, 21(5), 1223–1235. 15813932 10.1111/j.1460-9568.2005.03966.x

[brb3944-bib-0030] Szurhaj, W. , & Derambure, P. (2006). Intracerebral study of gamma oscillations in the human sensorimotor cortex. Progress in Brain Research, 159(1), 297. 17071239 10.1016/S0079-6123(06)59020-X

[brb3944-bib-0031] Szurhaj, W. , Labyt, E. , Bourriez, J. L. , Kahane, P. , Chauvel, P. , Mauguière, F. , & Derambure, P. (2006). Relationship between intracerebral gamma oscillations and slow potentials in the human sensorimotor cortex. European Journal of Neuroscience, 24(3), 947–954. 16930422 10.1111/j.1460-9568.2006.04876.x

[brb3944-bib-0032] Navalpakkam, V. , & Laurent, I. (2007). Search Goal Tunes Visual Features Optimally. Neuron, 53, 605–617. 17296560 10.1016/j.neuron.2007.01.018

[brb3944-bib-0033] Virués‐Ortega, J. , Buela‐Casal, G. , Garrido, E. , & Alcázar, B. (2004). Neuropsychological functioning associated with high‐altitude exposure. Neuropsychology Review, 14(4), 197–224. https://doi.org/10.1007/s11065-004-8159-4 15796116 10.1007/s11065-004-8159-4

[brb3944-bib-0034] Wang, Y. , Ma, H. , Fu, S. , Guo, S. , Yang, X. , Luo, P. , & Han, B. (2014). Long‐term exposure to high altitude affects voluntary spatial attention at early and late processing stages. Scientific Reports, 4, 4443.

[brb3944-bib-0035] Woodman, G. F. , & Luck, S. J. (1999). Electrophysiological measurement of rapid shifts of attention during visual search. Nature, 400(6747), 867. 10476964 10.1038/23698

[brb3944-bib-0036] Yan, X. (2014). Cognitive impairments at high altitudes and adaptation. High Altitude Medicine & Biology, 15(2), 141–145. 24949527 10.1089/ham.2014.1009

